# Increasing the library size in cDNA display by optimizing purification procedures

**DOI:** 10.1186/1480-9222-15-7

**Published:** 2013-05-22

**Authors:** Yuki Mochizuki, Shigefumi Kumachi, Koichi Nishigaki, Naoto Nemoto

**Affiliations:** 1 Graduate School of Science and Engineering, Saitama University, 255 Shimo-Okubo, Sakura-ku, Saitama 338-8570, Japan

**Keywords:** Directed evolution, *In vitro* protein selection, mRNA/cDNA display, Protein engineering, Puromycin

## Abstract

**Background:**

The library size is critical for selection in evolutionary molecular engineering (directed evolution). Although cDNA display has become a promising *in vitro* display technology by overcoming the instability of mRNA display, it is hindered by low yields. In this study, we improved the yield of cDNA display molecules by carefully examining each step of the preparation process.

**Findings:**

We found that steric hindrance of ribosomes binding to the mRNA-protein fusion molecules was interfering with biotin-streptavidin binding. Additionally, reducing buffer exchange by performing RNase digestion in the His-tag-binding buffer to release the cDNA display molecules improved their His-tag purification.

**Conclusion:**

Our optimized conditions have improved the yield of cDNA display molecules by more than 10 times over currently used methods, making cDNA display more practically available in evolutionary molecular engineering.

## Findings

For nearly two decades, evolutionary molecular engineering (directed evolution) has played a role as a complementary partner to rational protein design in protein engineering [1]. In evolutionary molecular engineering, the size of the library is crucial for selection efficiency. Recently, the productivity and versatility of *in vitro* display technologies have increased the size of libraries by using a cell-free translation system. For example, ribosome [2] and mRNA displays [3,4] have large libraries because the amount of mRNA-peptide/protein (genotype-phenotype) complex with a ribosome or puromycin is proportional to the input of mRNA (~10^12^/ml) in a cell-free translation system. In the case of mRNA display, ligation methods have been developed to synthesize mRNA-puromycin-linker practically [5-7]. However, essentially the lability of mRNA in both ribosome and mRNA displays has restricted the experimental selection conditions.

Thus, the cDNA display method was developed to improve the stability by converting mRNA to cDNA with a novel puromycin-linker [8]. This technology allows researchers to screen large combinatorial libraries against molecules on a cell surface (e.g. receptors) [9], and to use peptide libraries containing two or more disulfide bonds [10,11]. Although the cDNA display method was useful for *in vitro* peptide and protein selection, its productivity was hindered by the generation of mRNA/cDNA-protein fusion molecules; only around 0.1% of the initial mRNA was ligated to proteins with a puromycin-linker [8]. Recently, this efficiency has been improved to more than 1% by introduction of a novel puromycin-linker and minor modification of previous method [12,13]. However, the yield of cDNA display fusion molecules is still smaller than that of mRNA display fusion molecules (20–30%).

**Figure 1:**
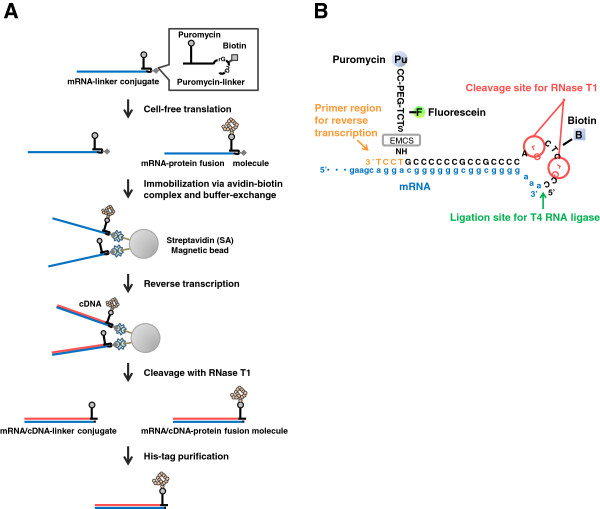
**Preparation method of cDNA display.** (**A**) Scheme of cDNA display preparation. The mRNA-linker conjugate was prepared by ligation of a puromycin-linker to the 3’-terminus of an mRNA coding B-domain of protein A (BDA). The mRNA-linker conjugate was translated by an *in vitro* translation reaction. The produced mRNA-protein fusion molecule and the remaining mRNA-linker conjugate were captured with SA-beads from the translation reaction mixture and reverse transcribed on the beads. The mRNA/cDNA-protein fusion molecule and the mRNA/cDNA molecule were released from the SA-beads by RNase T1 treatment. The mRNA/cDNA-protein fusion molecule was purified by the His-tag in the translated protein. (**B**) Schematic diagram of construct of short biotin segment puromycin-linker (SBP-linker). The SBP-linker construct comprises four parts: a ligation site for mRNA, a primer region for reverse transcription, a biotin moiety for the immobilization of the mRNA-linker conjugate on Streptavidin-beads, and two cleavage sites for RNase T1 to release the mRNA/cDNA-protein fusion molecule from the SA-beads. In addition, the SBP-linker includes puromycin (for the covalent linking of the expressed protein to mRNA) and fluorescein (for detection and quantification). The 3’-region of the mRNA is shown in lower case letters. [N-(6-maleimidcaproyloxy) succinimide] (EMCS) is bifunctional cross-linker used in the preparation of the SBP-linker.

The aims of this study were to investigate which processes during the preparation of cDNA display fusion molecules shown in Figure 1 cause its low yield, and to increase the yield by overcoming these problems.

**Figure 2:**
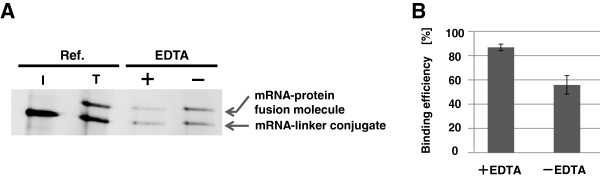
**Effect of EDTA treatment on the capturing of mRNA-protein fusions by SA-beads.** mRNA-protein fusion molecules in the translation reaction mixture with (+) or without (−) EDTA were incubated with SA-beads [the ratio of the biotin-binding capacity of the SA-beads to the total amount of puromycin-linker conjugate is 200 to 1]. (**A**) The remaining mRNA-protein fusion molecules in each translation reaction mixture were analyzed by 4% stacking–6% separating SDS PAGE containing 8 M urea. The input mRNA-linker conjugates and translation reaction mixture are shown in lane I and lane T, respectively. (**B**) Binding efficiencies are calculated by: [band intensity of the mRNA-protein fusion molecules in lane T] - [band intensity of the remaining mRNA-protein fusion molecules in each lane indicated by “+” or “–” EDTA].

**Figure 3:**
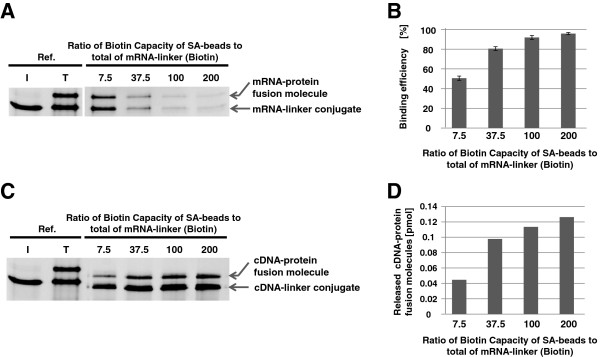
**Optimization of the amount of SA-beads to bind the mRNA-protein fusion molecules.** (**A**) mRNA-protein fusion was prepared from DNA template coding B domain of protein A (BDA) using SBP-linker as described Figure 1. Translation mixture was treated with EDTA same as Figure 2. Then the mixture was incubated with 0.0075–0.2 mg of SA-beads per 0.5 pmol of mRNA estimated from the amount in the ligation reaction [the ratio of the biotin-binding capacity of the SA-beads to the total amount of SBP-linker (containing a single biotin) is 7.5–200]. Inputted mRNA-linker conjugates (lane I), translation mixture (lane T), and remaining mRNA-protein fusion molecules in the translation mixture after incubation with different amounts of SA-beads were analyzed by 4% stacking–6% separating SDS-PAGE containing 8 M urea. (**B**) Binding efficiencies of each ratio were calculated by: [band intensity of the mRNA-protein fusion molecules in lane T] - [band intensity of the remaining mRNA-protein fusion molecules in each lane indicated by SA-beads]. Experiments were repeated 3 times. Error bars = standard deviation. (**C**) mRNA/cDNA-protein fusion molecules were prepared with each ratio of SA-beads. Reverse transcription (RT) was performed at 40°C for at least 10 min in 20 μL of the RT reaction mixture [50 mM Tris–HCl, pH 8.3, 75 mM KCl, 3 mM MgCl2, 50 mM dithiothreitol (DTT), 0.5 mM dNTP mix and 200 U of SuperScriptIII reverse transcriptase (Invitrogen)] . 20 U of RNase T1 (Ambion) and RNase H (Takara Bio Inc., Kyoto, Japan) were added to the RT reaction mixture and incubated at 37°C for 10 min. Released mRNA/cDNA-protein fusion molecules were confirmed by SDS-PAGE as the above. (**D**) The amount of cDNA-protein fusion molecules calculated by comparing the band intensity between the cDNA-protein fusion molecule of each lane and 0.5 pmol of mRNA-linker conjugate.

First, we determined the binding capacity of streptavidin-coated magnetic beads (SA-beads) (Dynabeads MyOne streptavidin C1 streptavidin magnetic beads, Invitrogen, Carlsbad, CA, USA) sufficient to collect most of the mRNA-protein fusion molecules from the rabbit reticulocyte lysate. In our previous study, SA-beads with a binding capacity of 360 pmol of biotinylated DNA primers were used for purifying of 48 pmol of mRNA-linker conjugates [8]. We thought this binding capacity should be sufficient for capturing this amount of biotinylated mRNA-protein fusion molecules, but the final yield of mRNA/cDNA-protein fusion molecule was much lower than expected. We speculated that ribosomes might strongly bind the mRNA-protein complex in the lysate before purification with the SA-beads. Indeed, we found that Ethylenediaminetetraacetic acid (EDTA) treatment for releasing ribosomes was effective to purify mRNA-protein fusion molecules from the translation mixture (Figure 2A, B). And in the present study, we found that SA-beads with a binding capacity 200 times the amount of biotinylated mRNA-protein fusion molecules were required to purify almost the all fusion molecules (Figure 3A, B). Similarly, the final amount of purified cDNA-protein fusion molecules also increased with increased SA-beads (Figure 3C, D). These results suggest that steric hindrance might interfere with biotin-streptavidin binding considerably.

**Figure 4:**
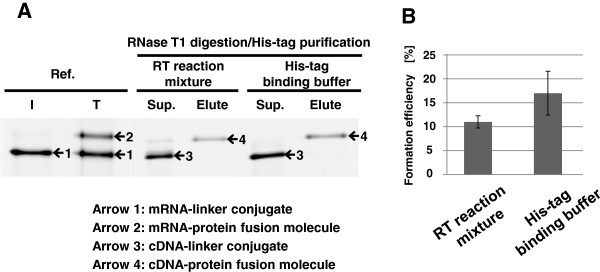
**Effect of His-tag-binding buffer in RNase T1 digestion and His-tag purification to recover mRNA/cDNA-protein fusion molecules from SA-beads.** (**A**) cDNA-protein fusion molecules were synthesized and released from the SA-beads by RNase T1 treatment in RT reaction mixture or His-tag-binding buffer (20 mM Sodium phosphate, pH 7.4, 500 mM NaCl, 5 mM imidazole, 0.05% Tween-20). cDNA-protein fusions in the each sample were purified with 20 μL of Ni-NTA beads (His Mag Sepharose Ni, GE Healthcare Bio-Sciences, Piscataway, NJ, USA) according to the attached instruction. Inputted mRNA-linker conjugates (lane I), translation mixture (lane T), each supernatant (Sup.) of Ni-NTA beads and eluate were analyzed by 4% stacking–6% separating SDS-PAGE containing 8 M urea. (**B**) Formation efficiencies of mRNA/cDNA-protein fusion molecules from mRNA-linker-conjugates were estimated by comparing the band intensities between the purified cDNA-protein fusion molecule of each lane and that of the mRNA-linker conjugate (lane I). Results are the mean of three independent experiments performed in duplicate. Error bars = standard deviation.

Second, we optimized the process from RNase T1 digestion to His-tag purification of cDNA display molecules shown in Figure 1A. In the cDNA display method, it is very important to separate mRNA/cDNA-protein fusion molecules from mRNA/cDNA-linker conjugates (which are not fused with its coding proteins) to reduce the background in the *in vitro* selection. Thus His-tag sequence (His X6) was incorporated to the C-terminal region of a coding protein. In this study we examined whether RNase T1 can digest the guanine base of the SBP-linker in the His-tag-binding buffer containing imidazole. If possible, buffer exchange for His-tag purification after RNase T1 digestion would be eliminated and so there could be no loss of cDNA display molecules. We found that RNase T1 worked well in the His-tag-binding buffer, and cDNA-protein fusion molecules were efficiently purified by His-tag purification without any buffer exchanges (Figure 4A, B). As a result, this modification increased the yield of mRNA/cDNA-protein fusion molecules by 1.5 times over the previous method. Moreover, this improvement allows us to save time and cost in the preparation of mRNA/cDNA-protein fusion molecules and to help make cDNA display technology easier to use.

One of the crucial problems with cDNA display is the low yield of cDNA-protein fusion molecules, which is less than 1% of input mRNA-linker conjugates [8,12]. In this paper, we identified that the mRNA-ribosome-protein complex may sterically hinder the biotin-streptavidin interaction between the puromycin-linker on the fusion molecules and the SA-beads. In addition, mRNA-protein fusion molecules without a ribosome could also interfere with the biotin-streptavidin interaction. Because of these reasons, more SA-beads than expected are required to purify most of the mRNA-protein fusion molecules. The addition of EDTA into the lysate after translation to remove the bound ribosome effectively increased the yield of cDNA display molecules. Furthermore, the simplification of His-tag purification after the release of cDNA display molecules from the SA-beads by performing RNase T1 digestion in the His-tag-binding buffer also increased the yield of cDNA display molecules. Finally we achieved 17% of final yield of cDNA display molecule based on the input mRNA-linker conjugates (Figure 4B), which is more than 10 times higher than in our previous study [8,12]. Additionally, we recently also succeeded in releasing cDNA display molecules from SA-beads by using Endonuclease V instead of RNase T1 by designing a new puromycin-linker [14]. Thus, we believe that this new linker and our currently optimized conditions will make cDNA display more useful and practical for in vitro protein selection.

## Abbreviations

SA-beads: Streptavidin-coated magnetic beads; BDA: B domain of protein A; SBP-linker: Short biotin segment puromycin-linker; EMCS: N-(6-maleimidcaproyloxy) succinimide; EDTA: Ethylenediaminetetraacetic acid; RT: Reverse transcription; DTT: Dithiothreitol; SDS-PAGE: Dodecyl sulfate–polyacrylamide gel electrophoresis.

## Competing interests

The authors declare that they have no competing interests.

## Authors’ contributions

YM and NN designed the study. YM and SK performed the experiments. YM, SK and NN analyzed the data and KN assisted with data interpretation. YM and NN contributed to the writing of the manuscript. All authors approved the manuscript.
